# Supplementary material for: Population-scale dietary interests during the COVID-19 pandemic
Source: Nat Commun. 2022 Feb 28;13:1073. doi: 10.1038/s41467-022-28498-z (PMC8885865; doi:10.1038/s41467-022-28498-z)
Supplement: Supplementary file 1 — Supplementary information [file 41467_2022_28498_MOESM1_ESM.pdf]

Supplementary material

1.1 Supplementary information, design choices and robustness checks

In Supplementary Figure 1, we present the detected mobility decreases and increases in the 18 countries. Supplementary Table 1 summarizes the descriptions of food categories and contains examples of popular foods in each category. We list all fitted coefficients and statistics of our main model in Supplementary Table 2. We provide correlation plots with Pearson correlation coefficient, instead of Spearman rank correlation coefficient in Supplementary Figure 2. Next, we provide our main results obtained with the RDD model with varying design choices and confirm that the qualitative interpretations of the effects remain stable under a number of robustness checks.

The impact of model order. We show our main results with a linear model in Supplementary Figures 3a and 3b, and in Supplementary Figures 4a and 4b with a constant model, instead of a quadratic model. While quadratic and linear models let us estimate the short-term effect (as illustrated in Figure 2), with the constant model we estimate the average effect in the entire period, from the discontinuity, until the bandwidth (K_2) weeks after discontinuity. The estimates of the effect with the constant model are then lower because the weeks when the effect diminishes are taken into account to calculate the average (see illustration in Supplementary Figure 7). While the nature and the amplitude of the estimated effect vary (i.e., whether the immediate short-term boost of average boost is captured), most of the conclusions are robust to this choice.

Additionally, for each category of food items, we fit a slightly different model pulling the interest volume across different countries similar to Equation 1, but with an added country-specific offset, that lets us measure effect across all countries. In Supplementary Table 3, as a robustness check, we show the food categories ranked by effect size pulled across countries, estimated with a constant, linear, and quadratic model. The rank between categories is strongly correlated (Spearman rank correlation 0.95 ($p = 3.7\times10^{-14}$) between constant and linear, 0.89 ($p = 3.2\times10^{-10}$) between constant and quadratic, and 0.94 ($p = 8.7 \times 10^{-14}$) between linear and quadratic models.

In Supplementary Figure 5, we show how the quadratic model fits the temporal evolution in the case of pastry and bakery category, in 18 countries. We also show linear (Supplementary Figure 6) and constant fit (Supplementary Figure 7) for comparison.

The impact of bandwidth. In Supplementary Figure 8, we study the impact of the choice of the bandwidth $\max(t_{\min}, t_{\max}) = 30$ and the choice of the degree of the model. We observe that for a sufficiently large bandwidth, all four models estimate a similar effect, and the choice of bandwidth does not matter as the estimates converge.

Modelling interest share. We show our main results with the same model, but the dependent variable being the weekly share of interest in Supplementary Figures 9a and 9b. This way, we control for overall increased interest in all categories. This analysis provides an alternative view. We see that the share of volume decreases significantly for foods whose growth is not proportional to the growth of the foods that experience major surges of interest.

1.2 Supplementary analyses

We perform supplementary analyses that support our main conclusions or provide complementary insights. In Supplementary Figure 10 we show short-term effects estimated with quadratic model, grouped by country. In each country, the gray line represents the overall country-specific short-term effect, that is the increase in interest in all food entities. Finally, we explore the effect of the second mobility decrease in Supplementary Figure 11, and we present the long-term effects in Supplementary Figure 12.

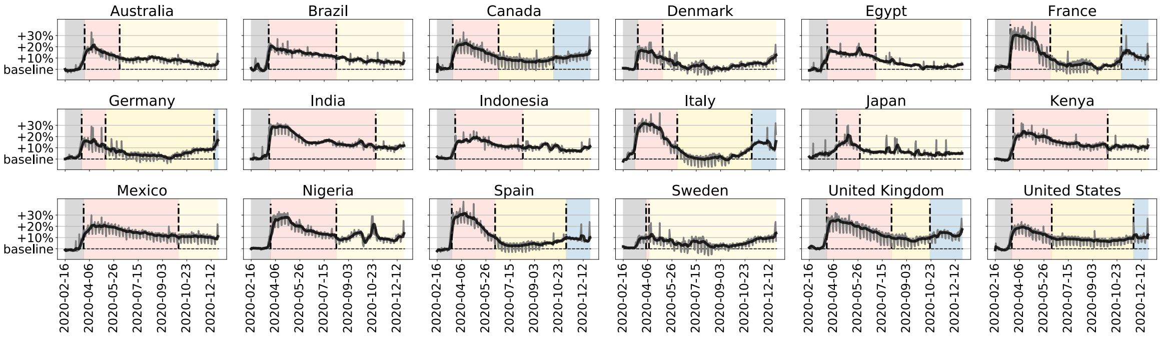

Supplementary Figure 1: Mobility in 18 studied countries. Mobility changepoints (mobility decrease, mobility increase, and the second mobility decrease in case it occurs) are marked with vertical dashed lines.

Supplementary Table 1: Summary of the 28 food entity categories. For each category, we present the category description, the number of entities in the category (Number), and category size (Size) that is the fraction of search interest covered by the category, on average, in the 18 studied countries in 2019 and 2020. Additionally, for each category, we show top 10 individual entities by the rank of the volume in average across 18 studied countries, in 2019 and 2020.

Category	Description	Number	Size
beef dish	food preparation based on beef	51	3.6%
Top 10 entities:	Hamburger, Beef, Steak, Meatball, Meatloaf, Beef Stroganoff, Fajita, Beef mince, Sirloin steak, Big Mac		
chicken dish	food preparation based on chicken	37	3.3%
Top 10 entities:	Chicken meat, Chicken nugget, Fried chicken, Chicken curry, Chicken soup Hendl, Butter chicken, Chicken tikka masala, Barbecue chicken, Cordon bleu		
pork dish	food preparation based on pork	45	2.1%
Top 10 entities:	Pork, Ham, Bacon, Hot dog, Pork chop, Gyro, Pork tenderloin, Pork belly, Pulled pork, Schnitzel		
lamb dish	food preparation based on lamb	17	0.5%
Top 10 entities:	Lamb and mutton, Shawarma, Doner kebab, Mechoui, Sfiha, Rogan josh, Cig kofte, Kokoretsi, Pasanda, Arrosticini		
fish dish	type of dish comprised of fish	57	1.8%
Top 10 entities:	Tuna, Caviar, Salmon, Cod, Squid, Sardine, Catfish, Crayfish, Tempura, Smoked salmon		
sausage	food usually made from ground meat with a skin around it	16	0.6%
Top 10 entities:	Sausage, Salami, Chorizo, Bratwurst, Mortadella, Black pudding, 'Nduja, Sujuk, Boudin, Andouille		
pasta, pizza and noodle dish	Italian food made from flour and water and shaped in different forms, usually cooked and served with a sauce, or a dish made with pasta, or other type of staple food made from some type of unleavened dough	95	6.9%
Top 10 entities:	Pizza, Pasta, Spaghetti, Lasagne, Noodle, Carbonara, Gnocchi, Macaroni, Penne, Ravioli		
potato dish	type of food based on potatoes	27	1.0%
Top 10 entities:	French fries, Mashed potato, Gratin, Baked potato, Tortilla de patatas, Potato, Potato pancake, Sunday roast, Tater Tots, Patatas bravas		
rice dish	a type of dish made of rice	49	3.8%
Top 10 entities:	Rice, Sushi, Risotto, Fried rice, Basmati, Paella, Biryani, Bento, Pilaf, White rice		
egg dish	a type of dish made of eggs	22	2.6%
Top 10 entities:	Egg, Boiled egg, Omelette, Quiche, Scrambled eggs, Poached egg, Frittata, Eggs Benedict, Deviled egg, Egg roll		
stew	combination of solid food ingredients that have been cooked in liquid and served in the resultant gravy	24	0.4%
Top 10 entities:	Stew, Ratatouille, Jambalaya, Dolma, Gumbo, Sambar, Cassoulet, Blanquette de veau, Irish stew, Bigos		
soup	primarily liquid food	55	2.7%
Top 10 entities:	Soup, Broth, Ramen, Miso, Pho, Hot pot, French onion soup, Goulash, Cream of mushroom soup, Minestrone		
bread and flatbread	staple food prepared from a dough	31	2.7%
Top 10 entities:	Bread, Pita, Bagel, Baguette, Sourdough, Naan, Pretzel, Focaccia, Bruschetta, White bread		
sandwich	two slices of bread with filling in between them	20	0.5%
Top 10 entities:	Sandwich, Panini, Corn dog, Croque-monsieur, Banh mi, BLT, Tuna fish sandwich, Peanut butter and jelly sandwich, Filet-O-Fish, Bocadillo		
salad	dish consisting of a mixture of small pieces of food, usually vegetables or fruit	24	1.7%
Top 10 entities:	Salad, Lettuce, Potato salad, Caesar salad, Pasta salad, Tabbouleh, Greek salad, Romaine lettuce, Insalata Caprese, Olivier salad		
cheese	yellow or white, creamy or solid food made from the pressed curds of milk	90	2.9%
Top 10 entities:	Cheese, Mozzarella, Cream cheese, Parmigiano-Reggiano, Ricotta, Feta, Cheddar cheese, Fondue, Mascarpone, Cottage cheese		
sauce	liquid, creaming or semi-solid food served on or used in preparing other foods	60	3.5%
Top 10 entities:	Sauces, Mayonnaise, Pesto, Dip, Mustard, Tomato sauce, Bechamel sauce, Bolognese sauce, Soy sauce, Gravy		
snack	portion of food, often smaller than a regular meal	20	1.8%
Top 10 entities:	Peanut, Popcorn, Hummus, Cashew, Tapas, Pistachio, Guacamole, Nachos, Cracker, Edamame		
vegetable and legume	edible plant or part of a plant, involved in cooking	85	9.5%
Top 10 entities:	Vegetable, Tomato, Sweet potato, Onion, Cucumber, Spinach, Eggplant, Cauliflower, Cabbage, Asparagus		
fruit	food, edible in the raw state	63	9.9%
Top 10 entities:	Apple, Lemon, Pineapple, Avocado, Grape, Mango, Watermelon, Cherry, Strawberry, Banana		
herb	plant part used for flavoring, food, medicine, or perfume	29	2.2%
Top 10 entities:	Lavender, Basil, Herb, Rosemary, Celery, Coriander, Parsley, Eucalyptus, Peppermint, Liquorice		
spice	dried seed, fruit, root, bark, or vegetable substance primarily used for flavoring, coloring or preserving food	38	5.1%
Top 10 entities:	Garlic, Table salt, Chili pepper, Ginger, Spice, Turmeric, Vanilla, Cinnamon, Common Fig, Black pepper		
soft drink	non-alcoholic drink, often carbonated (sparkling)	27	2.1%
Top 10 entities:	Coca-Cola, Juice, Soft drink, Cola, Lemonade, Orange juice, Tonic water, Energy drink, Iced tea, Apple juice		
wine, beer and liquor	alcoholic drink, alcoholic drink typically made from grapes, or alcoholic beverage that is produced by distilling	46	6.6%
Top 10 entities:	Wine, Vodka, Beer, Alcoholic beverage, Rum, Gin, Champagne, Tequila, Red Wine, Sake		
cocktail	alcoholic mixed drink	142	1.3%
Top 10 entities:	Cocktail, Mojito, Martini, Sour, Margarita, Gin and tonic, Piña colada, Mimosa, Spritz, Bloody Mary		
pie	baked dish usually made of a pastry dough casing, containing a filling of various sweet or savoury ingredients	20	1.3%
Top 10 entities:	Pie, Tart, Apple pie, Cottage pie, Pumpkin pie, Borek, Tarte Tatin, Meat pie, Banoffee pie, Lemon meringue pie		
pastry and bakery product	various baked products made of dough	40	1.5%
Top 10 entities:	Baking powder, Pastry, Baker's yeast, Puff pastry, Brioche, Samosa, Filo, Ice cream cone, Choux pastry, Cannoli		
dessert	course that concludes a meal; usually very sweet	202	18.2%
Top 10 entities:	Cake, Chocolate, Ice cream, Honey, Pancake, Biscuit, Cookie, Doughnut, Cupcake, Chocolate brownie		

Draft

Supplementary Table 2: Main quadratic model: fitted coefficient alpha and R^2 statistic. Entities Recipe, Food delivery, Restaurant, and Picnic mark the sets of entities described in Figure 5a.

Access mode	AU	BR	CA	DE	DK	EG	ES	FR	GB	ID	IN	IT	JP	KE	MX	NG	SE	US
Recipe	0.34(0.9)	0.46(0.9)	0.57(0.87)	0.43(0.77)	0.29(0.77)	0.28(0.74)	0.84(0.9)	0.73(0.81)	0.78(0.94)	0.64(0.69)	1.03(0.95)	0.77(0.87)	-0.08(0.93)	0.73(0.88)	0.58(0.93)	0.4(0.79)	0.09(0.78)	0.6(0.91)
Food delivery	0.78(0.9)	0.7(0.93)	0.97(0.95)	0.15(0.85)	1.05(0.9)	-0.46(0.15)	-0.09(0.66)	0.96(0.57)	0.37(0.86)	1.4(0.34)	0.37(0.24)	0.95(0.7)	1.38(0.95)	-0.34(0.18)	0.4(0.65)	0.02(0.18)	-0.35(0.74)	1.11(0.96)
Restaurant	-0.78(0.86)	-0.23(0.88)	-0.68(0.87)	-0.98(0.84)	-0.7(0.75)	-0.75(0.89)	-1.65(0.93)	-1.7(0.92)	-0.95(0.84)	-0.71(0.89)	-0.49(0.97)	-1.45(0.88)	-0.62(0.92)	-0.0(0.79)	-0.54(0.93)	-0.99(0.67)	-0.13(0.76)	-0.39(0.82)
Picnic	0.1(0.7)	0.16(0.85)	0.24(0.97)	0.44(0.7)	0.19(0.55)	-0.9(0.36)	-0.25(0.75)	-0.58(0.77)	0.92(0.87)	0.26(0.59)	-0.36(0.92)	-0.6(0.72)	-0.21(0.78)	0.92(0.35)	-0.11(0.49)	0.97(0.35)	0.51(0.7)	-0.06(0.84)
Food categories	AU	BR	CA	DE	DK	EG	ES	FR	GB	ID	IN	IT	JP	KE	MX	NG	SE	US
beef dish	0.16(0.79)	0.35(0.82)	0.24(0.63)	0.26(0.4)	0.16(0.39)	-0.0(0.28)	0.59(0.7)	0.38(0.68)	0.55(0.77)	0.2(0.3)	0.23(0.64)	0.17(0.39)	-0.28(0.92)	0.21(0.48)	0.2(0.66)	0.32(0.37)	0.12(0.63)	0.41(0.66)
bread and flatbread	0.39(0.91)	0.6(0.96)	1.08(0.9)	0.48(0.93)	0.69(0.88)	0.21(0.39)	1.41(0.91)	1.36(0.9)	0.67(0.96)	0.66(0.84)	0.64(0.8)	1.37(0.86)	-0.16(0.91)	0.42(0.71)	0.13(0.92)	0.61(0.76)	-0.04(0.85)	0.84(0.92)
cheese	0.37(0.74)	0.45(0.85)	0.35(0.68)	0.18(0.8)	0.26(0.52)	0.56(0.58)	0.64(0.65)	0.44(0.6)	0.62(0.76)	0.62(0.73)	0.6(0.89)	0.41(0.64)	0.04(0.87)	1.0(0.51)	0.62(0.66)	-0.33(0.45)	0.12(0.57)	0.27(0.66)
chicken dish	0.25(0.84)	0.28(0.91)	0.3(0.81)	0.31(0.77)	0.19(0.65)	0.74(0.31)	0.77(0.87)	0.52(0.67)	0.57(0.82)	0.59(0.43)	0.38(0.79)	0.5(0.81)	0.04(0.92)	0.76(0.6)	0.34(0.91)	0.5(0.61)	0.01(0.72)	0.29(0.83)
cocktail	0.25(0.73)	0.35(0.73)	0.29(0.8)	0.06(0.84)	-0.18(0.79)	-0.52(0.45)	0.09(0.74)	0.26(0.77)	0.7(0.72)	0.33(0.53)	-0.23(0.4)	0.5(0.79)	0.13(0.56)	-0.56(0.39)	-0.1(0.51)	-1.19(0.59)	0.02(0.66)	0.31(0.66)
dessert	0.44(0.79)	0.41(0.89)	0.56(0.83)	0.34(0.71)	0.32(0.69)	0.36(0.63)	0.99(0.9)	0.74(0.81)	1.01(0.85)	0.42(0.77)	0.51(0.76)	0.66(0.85)	0.08(0.63)	0.46(0.87)	0.27(0.88)	0.14(0.91)	-0.02(0.66)	0.37(0.72)
egg dish	0.82(0.74)	0.24(0.61)	0.23(0.58)	0.29(0.66)	0.14(0.47)	-0.02(0.56)	0.73(0.79)	0.58(0.81)	0.55(0.81)	0.68(0.79)	0.55(0.9)	0.33(0.73)	0.05(0.89)	0.27(0.66)	0.34(0.82)	0.55(0.75)	-0.37(0.55)	0.21(0.56)
fish dish	0.12(0.44)	0.36(0.38)	0.14(0.66)	0.24(0.5)	0.33(0.22)	0.52(0.15)	0.65(0.55)	0.27(0.57)	0.29(0.79)	0.19(0.69)	0.08(0.68)	0.11(0.31)	0.03(0.94)	0.5(0.25)	0.38(0.61)	0.35(0.46)	0.35(0.33)	0.16(0.85)
fruit	0.36(0.87)	0.21(0.8)	0.33(0.9)	0.2(0.79)	0.05(0.61)	0.21(0.77)	0.6(0.83)	0.64(0.77)	0.65(0.94)	0.21(0.63)	0.28(0.83)	0.33(0.78)	-0.11(0.9)	0.55(0.91)	0.35(0.93)	0.27(0.79)	0.09(0.78)	0.19(0.89)
herb	0.03(0.78)	0.05(0.95)	0.21(0.91)	0.16(0.93)	0.45(0.66)	0.39(0.48)	0.16(0.81)	0.19(0.87)	0.58(0.91)	-0.03(0.54)	0.17(0.92)	0.27(0.76)	0.05(0.89)	0.57(0.57)	-0.18(0.71)	0.25(0.75)	0.31(0.84)	0.14(0.92)
lamb dish	0.18(0.82)	0.61(0.71)	-0.04(0.35)	0.05(0.75)	0.13(0.22)	-0.4(0.54)	-0.31(0.08)	-0.32(0.4)	0.31(0.59)	0.09(0.21)	-0.14(0.47)	-0.37(0.5)	-0.32(0.9)	2.51(0.28)	-0.11(0.18)	0.29(0.65)	0.17(0.44)	0.11(0.34)
pasta, pizza & noodle dish	0.22(0.8)	0.43(0.78)	0.28(0.82)	0.17(0.63)	0.25(0.4)	0.43(0.76)	0.66(0.77)	0.34(0.65)	0.37(0.91)	0.58(0.88)	0.5(0.88)	0.61(0.72)	-0.17(0.91)	0.52(0.76)	0.3(0.77)	0.44(0.69)	0.16(0.61)	0.27(0.68)
pastry & bakery product	0.52(0.86)	0.91(0.93)	1.26(0.81)	0.96(0.87)	0.43(0.55)	1.14(0.51)	1.45(0.86)	1.32(0.82)	1.1(0.93)	0.34(0.49)	1.05(0.74)	1.08(0.84)	0.72(0.63)	1.08(0.6)	-0.0(0.32)	0.66(0.75)	0.2(0.44)	1.06(0.83)
pie	0.37(0.88)	0.49(0.87)	0.8(0.79)	0.3(0.45)	0.58(0.5)	0.6(0.36)	1.14(0.79)	0.74(0.77)	0.75(0.83)	0.04(0.54)	0.31(0.44)	0.84(0.84)	0.13(0.75)	0.59(0.4)	0.65(0.63)	0.57(0.64)	0.13(0.86)	0.66(0.75)
pork dish	0.19(0.85)	0.32(0.88)	0.35(0.59)	0.23(0.43)	0.31(0.28)	0.7(0.15)	0.42(0.69)	0.34(0.76)	0.56(0.85)	0.58(0.52)	0.19(0.83)	0.4(0.53)	0.04(0.92)	1.08(0.45)	0.49(0.81)	0.53(0.35)	0.08(0.63)	0.34(0.61)
potato dish	0.33(0.72)	0.46(0.87)	0.53(0.58)	0.47(0.77)	0.01(0.59)	0.88(0.18)	0.61(0.72)	0.59(0.82)	0.66(0.78)	0.57(0.67)	0.63(0.93)	0.47(0.83)	0.05(0.82)	0.34(0.48)	1.11(0.67)	2.26(0.29)	0.04(0.7)	0.44(0.73)
rice dish	0.17(0.75)	0.4(0.74)	0.16(0.66)	0.1(0.73)	0.24(0.5)	0.34(0.43)	0.63(0.68)	0.37(0.75)	0.28(0.87)	0.42(0.6)	0.61(0.89)	0.21(0.54)	-0.06(0.92)	0.49(0.56)	0.32(0.85)	0.4(0.68)	0.15(0.67)	0.23(0.85)
salad	0.18(0.84)	0.11(0.61)	0.04(0.9)	0.16(0.81)	-0.08(0.79)	0.32(0.37)	0.54(0.89)	0.41(0.85)	0.6(0.81)	0.44(0.48)	0.35(0.39)	0.24(0.8)	-0.02(0.93)	0.14(0.23)	0.24(0.64)	-0.67(0.29)	0.28(0.8)	0.02(0.85)
sandwich	0.25(0.48)	0.04(0.4)	-0.13(0.5)	0.17(0.31)	-0.09(0.28)	0.09(0.38)	0.26(0.25)	0.25(0.46)	0.14(0.49)	0.47(0.39)	0.33(0.69)	0.34(0.41)	0.09(0.91)	-5.08(0.28)	0.18(0.5)	0.53(0.13)	0.62(0.31)	-0.18(0.36)
sauce	0.38(0.84)	0.43(0.86)	0.31(0.8)	0.26(0.72)	0.36(0.6)	0.56(0.56)	0.71(0.81)	0.49(0.69)	0.6(0.86)	0.62(0.69)	0.71(0.9)	0.52(0.57)	0.19(0.93)	0.91(0.62)	0.46(0.84)	-0.42(0.59)	0.09(0.76)	0.33(0.85)
sausage	0.33(0.72)	0.29(0.77)	0.18(0.54)	-0.01(0.47)	0.15(0.22)	0.16(0.12)	0.77(0.73)	0.36(0.75)	0.55(0.8)	0.44(0.56)	0.81(0.56)	0.38(0.44)	0.16(0.77)	2.41(0.22)	0.27(0.75)	0.35(0.45)	-0.32(0.33)	0.34(0.77)
snack	0.28(0.56)	0.3(0.75)	0.26(0.78)	0.02(0.64)	-0.11(0.34)	-0.04(0.56)	0.13(0.45)	0.37(0.67)	0.57(0.72)	0.01(0.38)	0.43(0.8)	0.18(0.58)	0.32(0.86)	-0.29(0.49)	0.08(0.39)	0.06(0.46)	0.28(0.24)	0.32(0.72)
soft drink	0.27(0.54)	-0.05(0.59)	-0.02(0.48)	0.09(0.82)	-0.04(0.46)	0.22(0.54)	0.47(0.61)	0.04(0.57)	0.32(0.72)	0.18(0.23)	-0.13(0.64)	0.53(0.41)	0.09(0.86)	-0.06(0.3)	0.15(0.49)	0.36(0.51)	-0.02(0.45)	-0.02(0.56)
soup	-0.11(0.92)	0.5(0.68)	0.27(0.91)	0.12(0.87)	0.22(0.85)	0.19(0.2)	0.5(0.74)	0.34(0.88)	0.29(0.94)	0.46(0.38)	0.34(0.63)	0.23(0.83)	-0.33(0.91)	-0.31(0.3)	0.31(0.56)	0.44(0.67)	-0.0(0.8)	0.25(0.96)
spice	0.28(0.84)	0.14(0.94)	0.27(0.85)	0.09(0.78)	0.03(0.57)	0.17(0.5)	0.49(0.78)	0.45(0.77)	0.49(0.85)	0.2(0.84)	0.42(0.95)	0.09(0.34)	0.07(0.9)	0.26(0.88)	0.16(0.89)	0.34(0.85)	0.03(0.52)	0.29(0.82)
stew	0.23(0.86)	0.55(0.58)	0.48(0.89)	0.41(0.52)	0.59(0.54)	-1.67(0.08)	0.57(0.78)	0.08(0.84)	0.43(0.87)	0.37(0.75)	0.55(0.83)	0.39(0.3)	0.18(0.8)	1.71(0.29)	0.49(0.25)	0.26(0.47)	0.77(0.28)	0.61(0.94)
vegetable and legume	0.29(0.9)	0.21(0.94)	0.28(0.86)	0.22(0.71)	0.34(0.46)	-0.02(0.62)	0.51(0.86)	0.69(0.79)	0.57(0.89)	0.4(0.74)	0.53(0.92)	0.27(0.73)	0.11(0.93)	0.57(0.85)	0.4(0.81)	0.26(0.6)	0.12(0.66)	0.3(0.84)
wine, beer and liquor	0.13(0.68)	0.19(0.85)	0.1(0.59)	0.02(0.82)	-0.04(0.68)	0.19(0.82)	0.06(0.64)	-0.29(0.78)	0.33(0.78)	-0.09(0.52)	0.18(0.55)	-0.16(0.53)	0.04(0.86)	0.87(0.56)	0.33(0.52)	0.3(0.27)	-0.13(0.7)	0.13(0.45)

Draft

Supplementary Table 3: The impact of model order. Food categories, ranked by short-term effect sizes in decreasing order, estimated with a constant, linear, and quadratic model.

Rank	Constant model	Linear model	Quadratic model
1	pastry and bakery product	pastry and bakery product	pastry and bakery product
2	pie	pie	bread and flatbread
3	dessert	bread and flatbread	potato dish
4	sauce	potato dish	pie
5	potato dish	dessert	dessert
6	bread and flatbread	sauce	cheese
7	chicken dish	chicken dish	sauce
8	stew	cheese	chicken dish
9	egg dish	vegetable and legume	pork dish
10	vegetable and legume	egg dish	sausage
11	cheese	pork dish	stew
12	fruit	pasta, pizza and noodle dish	pasta, pizza and noodle dish
13	herb	fruit	egg dish
14	spice	stew	vegetable and legume
15	sausage	rice dish	fruit
16	rice dish	spice	rice dish
17	pasta, pizza and noodle dish	herb	fish dish
18	fish dish	sausage	beef dish
19	pork dish	fish dish	spice
20	salad	snack	herb
21	snack	beef dish	soup
22	beef dish	salad	snack
23	sandwich	sandwich	salad
24	soft drink	soup	lamb dish
25	soup	soft drink	soft drink
26	lamb dish	wine, beer and liquor	wine, beer and liquor
27	wine, beer and liquor	cocktail	cocktail
28	cocktail	lamb dish	sandwich

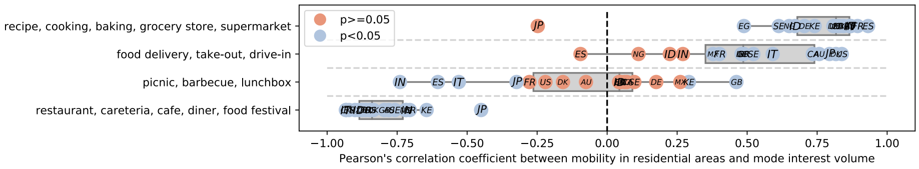

(a)

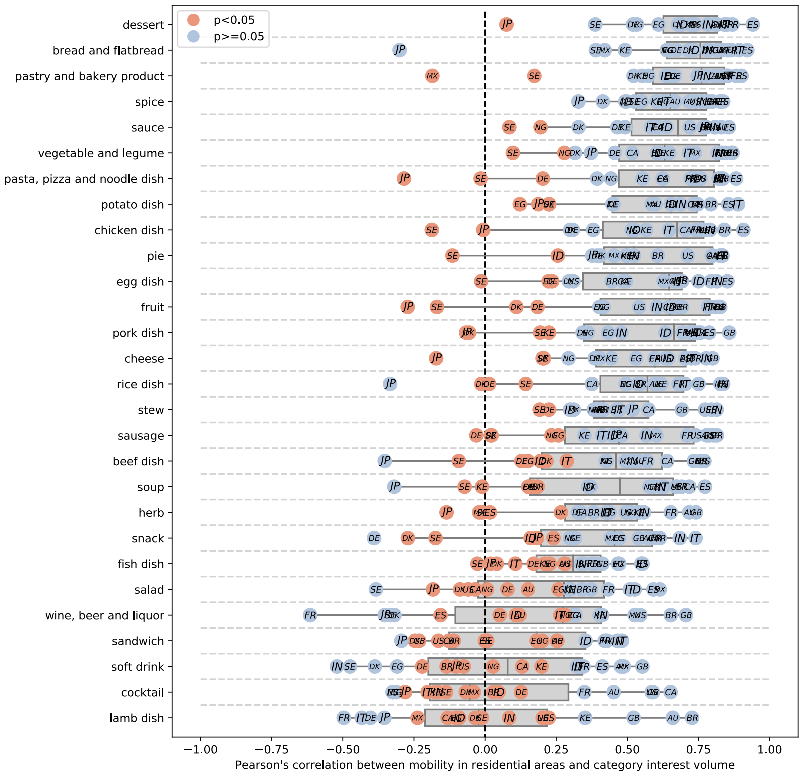

(b)

Supplementary Figure 2: Pearson's correlation coefficient between mobility and interest volume. In (a), correlation for categories of food entities, and in (b), for ways of accessing food. For each group, $n = 18$ values represent correlation coefficient (calculated based on $n = 46$ samples corresponding to weeks of 2020). The boxplot summarizes the value across 18 countries. Significant correlations ($p < 0.05$), according to a two-sided hypothesis test whose null hypothesis is that interest and mobility are uncorrelated, are marked in blue, and not significant in orange. No adjustments for multiple comparisons are made. Boxplots represent the 50th (center line), 25th and 75th percentile (box limits). The whiskers extend to the minimum and maximum values but no further than 1.5 times IQR.

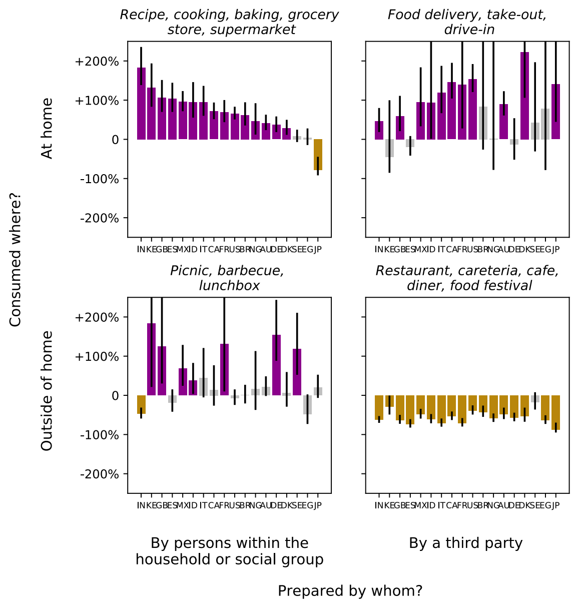

(a)

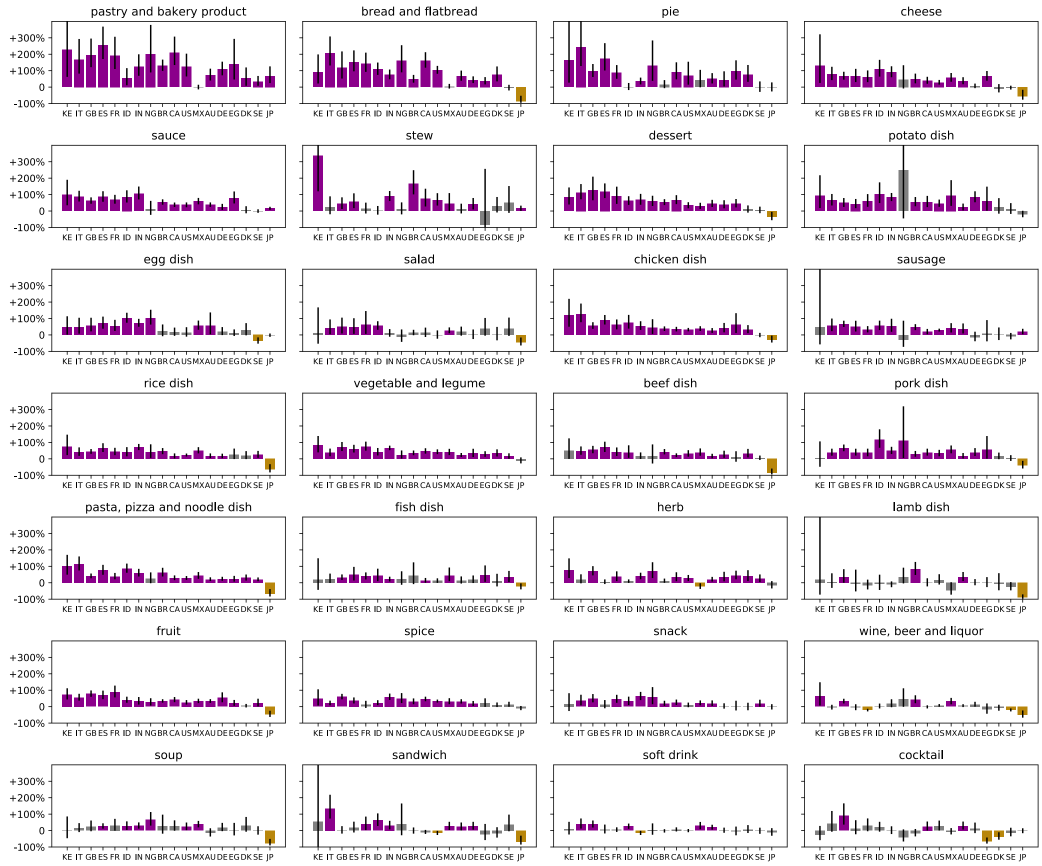

(b)

Supplementary Figure 3: Short-term effects estimated with a linear model. For each country ($n = 18$), for each food access mode (in (a), $n = 4$) and food category (in (b), $n = 28$), model (Eq. 1) is fitted on $n = 82$ samples. Bars represent effect estimates (coefficient α estimated with our RDD-based model). Error bars mark 95% confidence intervals. Purple marks significant positive ($p < 0.05$), yellow significant negative ($p < 0.05$), and grey marks non-significant effects.

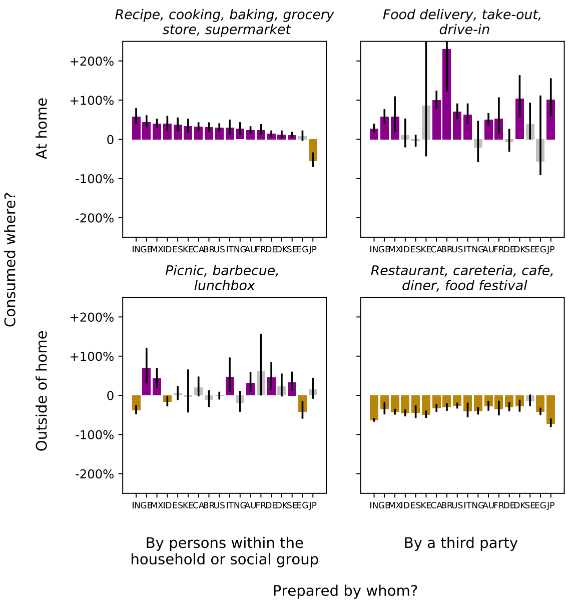

(a)

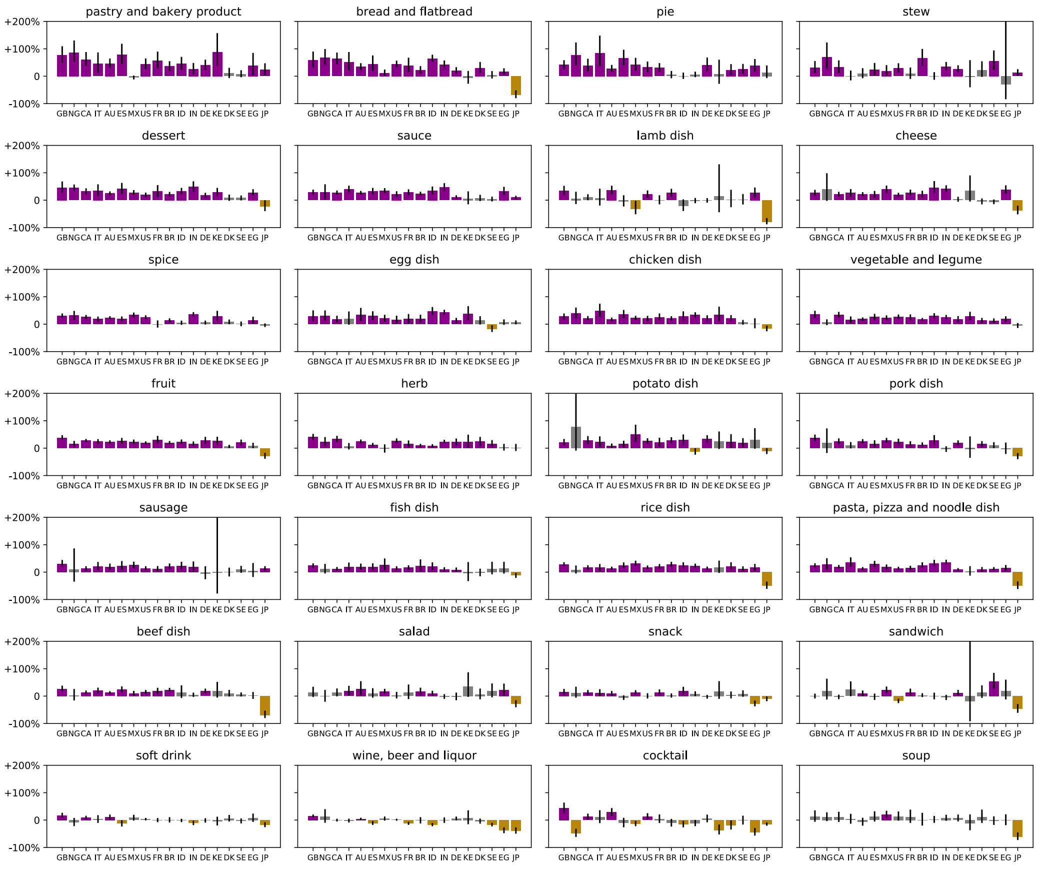

(b)

Supplementary Figure 4: Short-term effects estimated with a constant model. For each country ($n = 18$), for each food access mode (in (a), $n = 4$) and food category (in (b), $n = 28$), model (Eq. 1) is fitted on $n = 82$ samples. Bars represent effect estimates (coefficient α estimated with our RDD-based model). Error bars mark 95% confidence intervals. Purple marks significant positive ($p < 0.05$), yellow significant negative ($p < 0.05$), and grey marks non-significant effects.

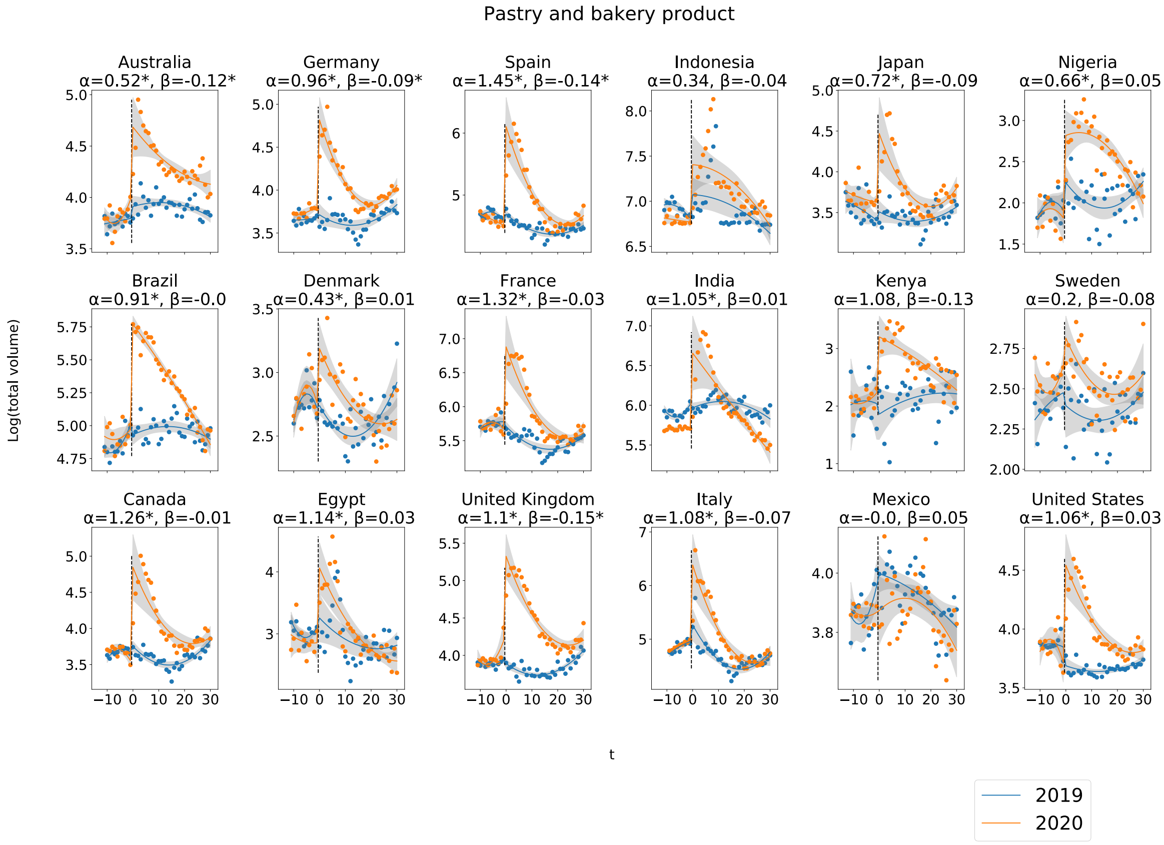

Supplementary Figure 5: Example of the quadratic model fit. On x-axis weeks, relative to the week of mobility decrease, on y-axis the interest volume. Error bars mark 95% confidence intervals of the model fit. α and β are fitted coefficients. Note the varying y-scales.

Draft

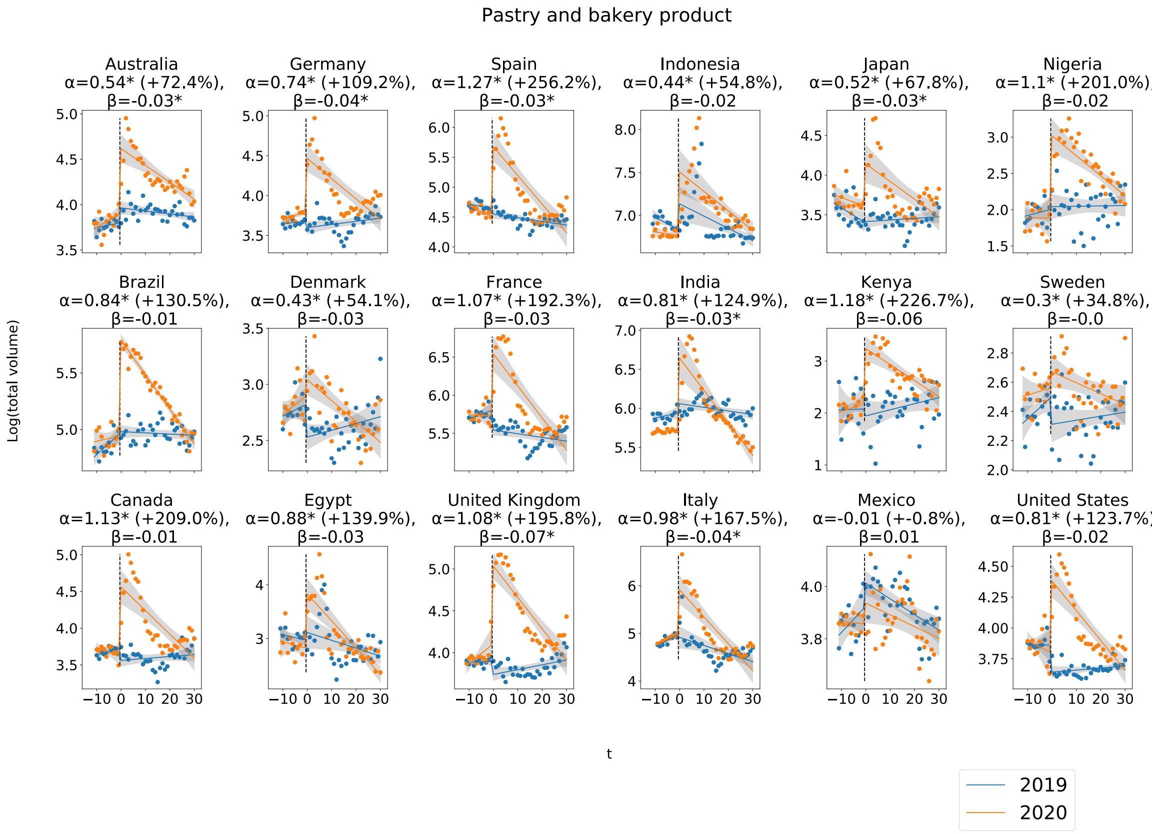

Supplementary Figure 6: Example of the linear model fit. On x-axis weeks, relative to the week of mobility decrease, on y-axis the interest volume. Error bars mark 95% confidence intervals of the model fit. α and β are fitted coefficients. Note the varying y-scales.

Draft

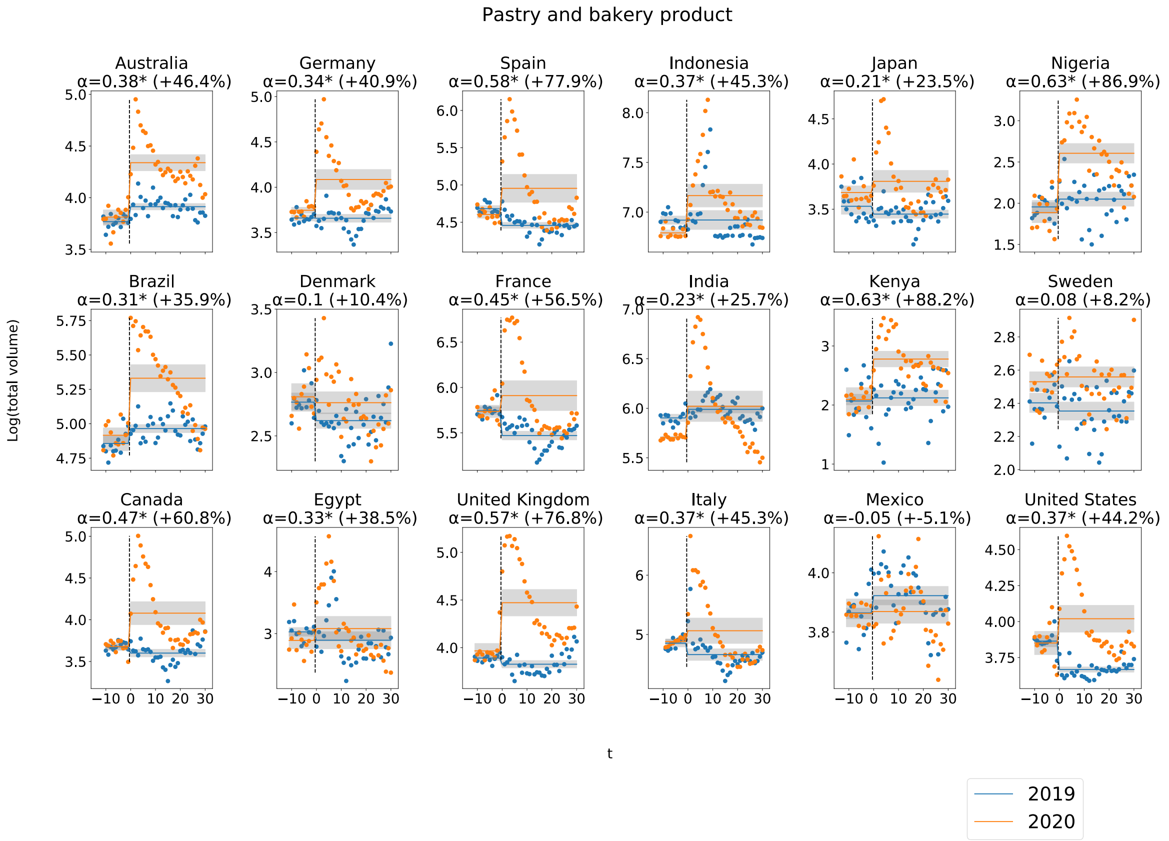

Supplementary Figure 7: Example of the constant model fit. On x-axis weeks, relative to the week of mobility decrease, on y-axis the interest volume. Error bars mark 95% confidence intervals of the model fit. α and β are fitted coefficients. Note the varying y-scales.

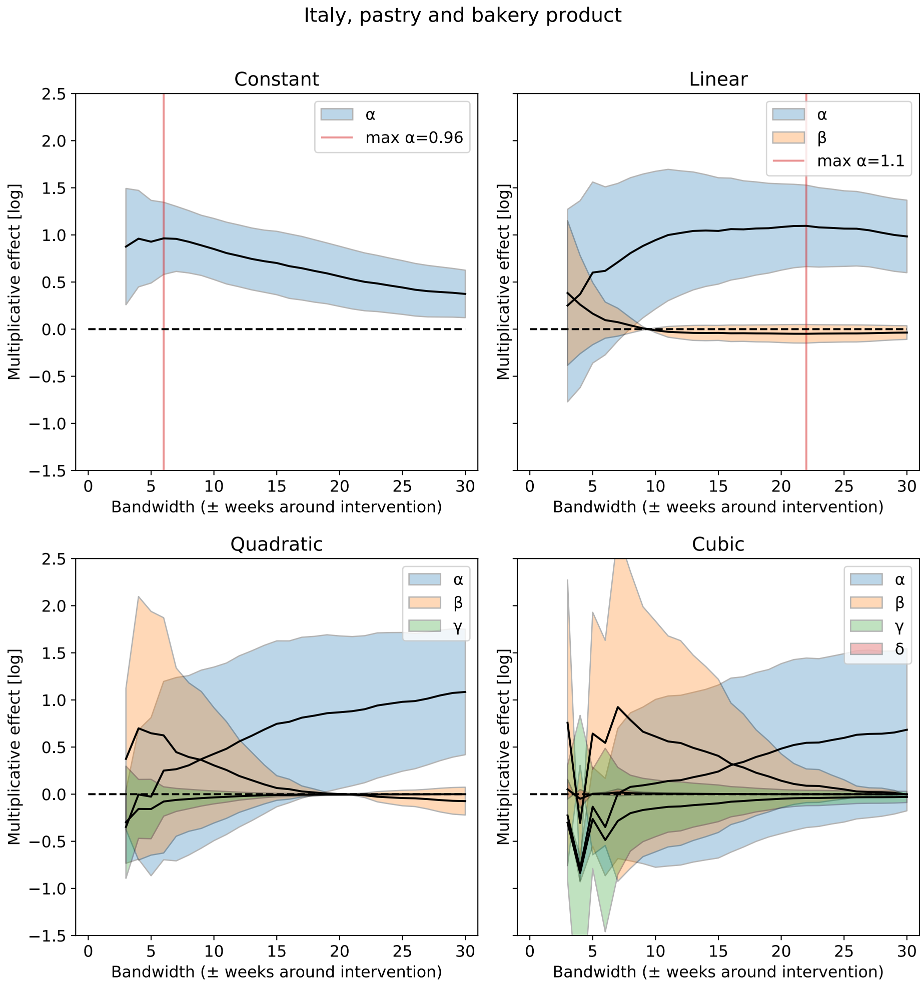

Supplementary Figure 8: Estimating the impact of the bandwidth (on x axis) on the fitted coefficients for constant, linear, quadratic, and cubic model. Error bars mark 95% confidence intervals of the fitted coefficients.

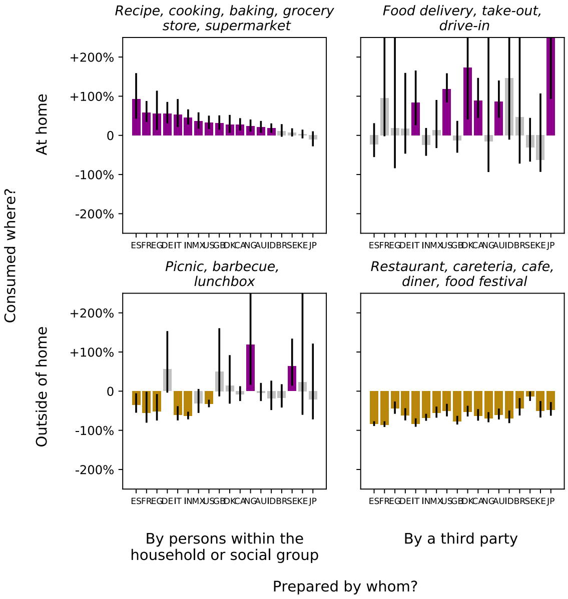

(a)

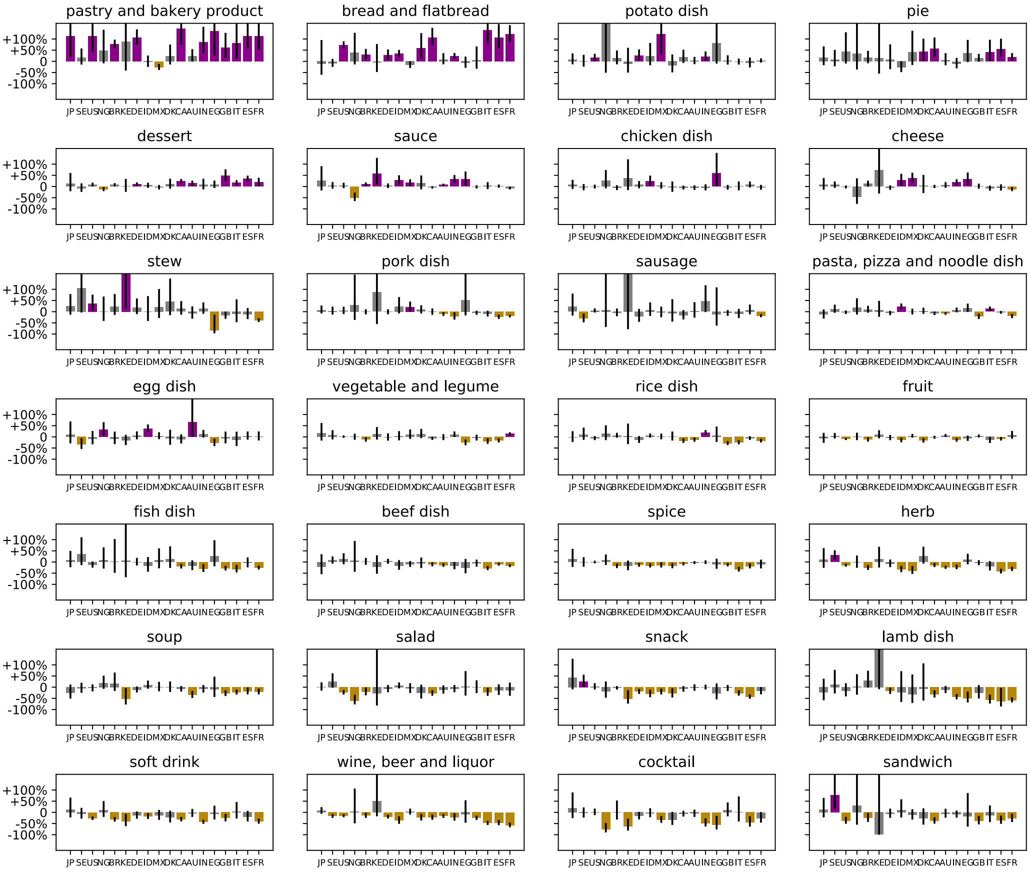

(b)

Supplementary Figure 9: Short-term effects on the share of interest, estimated with a quadratic model. For each country ($n = 18$), for each food access mode (in (a), $n = 4$) and food category (in (b), $n = 28$), model (Eq. 1) is fitted on $n = 82$ samples. Bars represent effect estimates (coefficient α estimated with our RDD-based model). Error bars mark 95% confidence intervals. Purple marks significant positive ($p < 0.05$), yellow significant negative ($p < 0.05$), and grey marks non-significant effects.

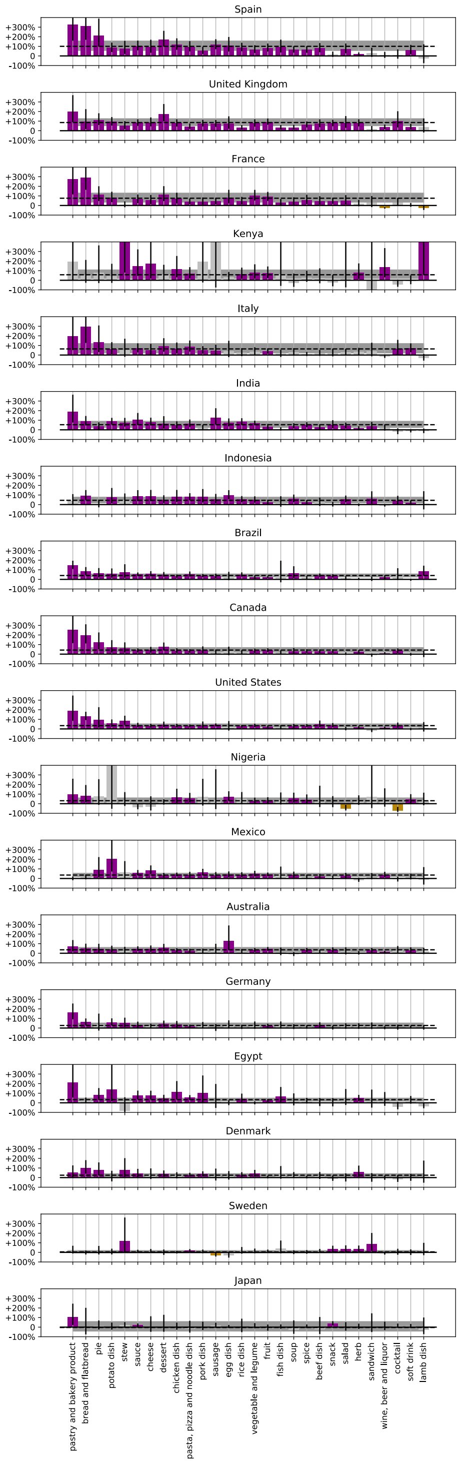

Supplementary Figure 10: Short-term effects across food categories, grouped by country. For each country ($n = 18$), for each food category ($n = 28$), model (Eq. 1) is fitted on $n = 82$ samples. Bars represent effect estimates (coefficient α estimated with our RDD-based model). Error bars mark 95% confidence intervals. Purple marks significant positive ($p < 0.05$), yellow significant negative ($p < 0.05$), and grey marks non-significant effects. The gray band marks the 95% CI of the effect on the country-specific total interest in all food entities.

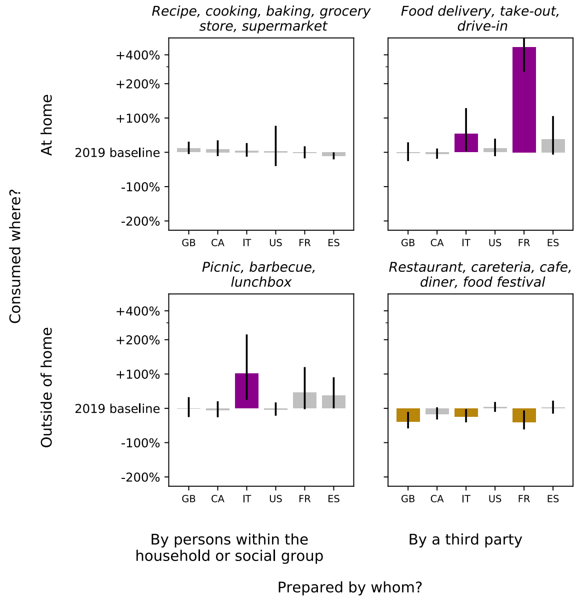

(a)

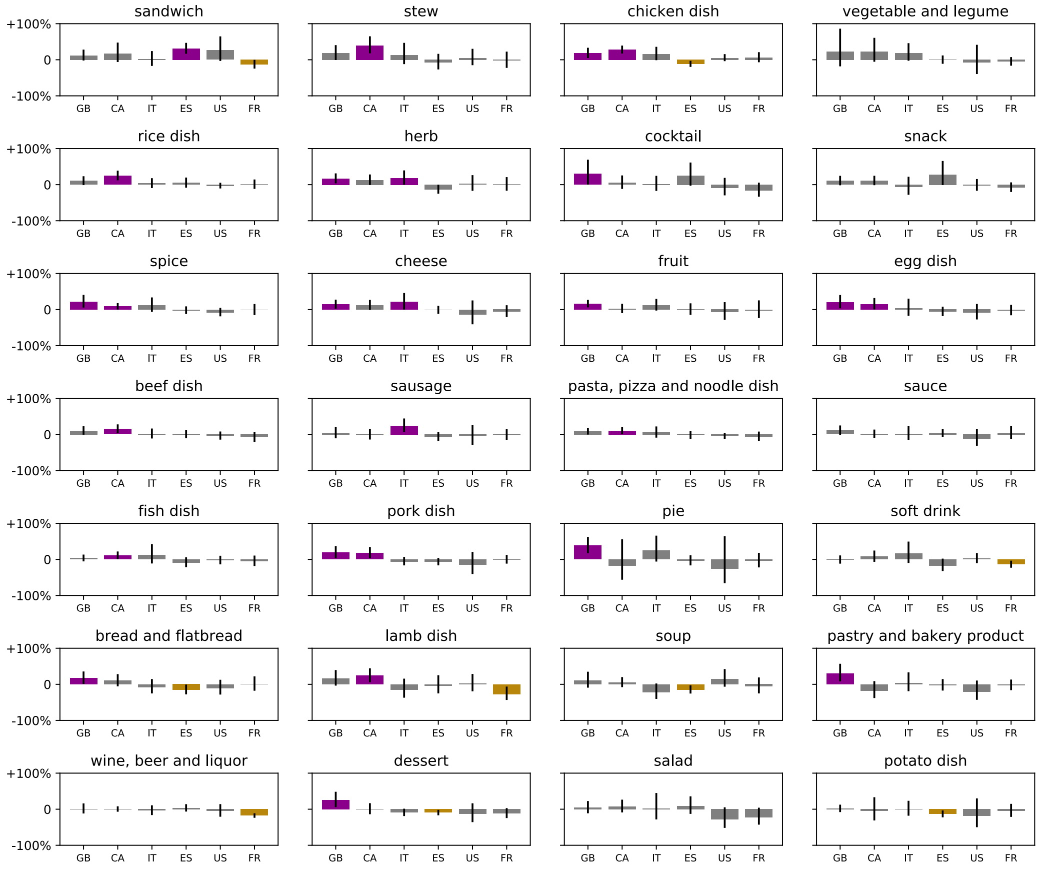

(b)

Supplementary Figure 11: Short-term effects estimated with a quadratic model, in the second wave. For each country ($n = 6$), for each food access mode (in (a), $n = 4$) and food category (in (b), $n = 28$), model (Eq. 1) is fitted on $n = 82$ samples. Bars represent effect estimates (coefficient α estimated with our RDD-based model). Error bars mark 95% confidence intervals. Purple marks significant positive ($p < 0.05$), yellow significant negative ($p < 0.05$), and grey marks non-significant effects.

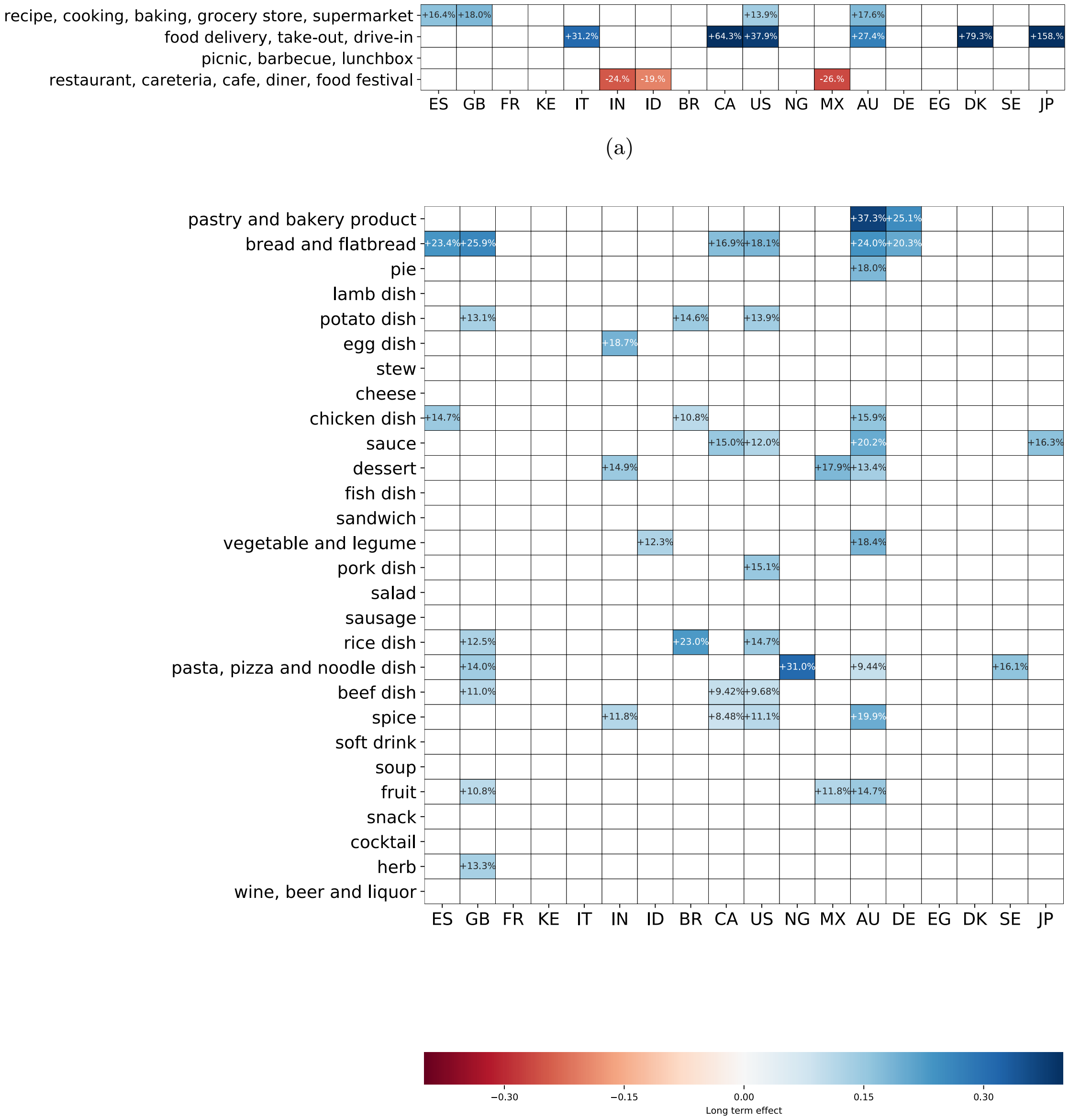

(a)

(b)

Supplementary Figure 12: Long-term effect of mobility decrease on food interests. In case the interest did not go back to normal within the 30 weeks after the mobility decrease, we measure how elevated the interest remains at the end of the modelled period, 30 weeks after mobility decrease, compared to the interest in 2019. White marks absence of long term effect when the interest eventually comes back to normal.
