# Supplementary material for: Population-scale dietary interests during the COVID-19 pandemic
Source: Nat Commun. 2022 Feb 28;13:1073. doi: 10.1038/s41467-022-28498-z (PMC8885865; doi:10.1038/s41467-022-28498-z)
Supplement: Supplementary file 2 — Reporting Summary [file 41467_2022_28498_MOESM2_ESM.pdf]

nature portfolio

Corresponding author(s): Kristina Gligoric

Last updated by author(s): Jan 21, 2022

Reporting Summary

Nature Portfolio wishes to improve the reproducibility of the work that we publish. This form provides structure for consistency and transparency in reporting. For further information on Nature Portfolio policies, see our Editorial Policies and the Editorial Policy Checklist.

Statistics

For all statistical analyses, confirm that the following items are present in the figure legend, table legend, main text, or Methods section.

n/a	Confirmed	
☐	☒	The exact sample size (*n*) for each experimental group/condition, given as a discrete number and unit of measurement
☐	☒	A statement on whether measurements were taken from distinct samples or whether the same sample was measured repeatedly
☐	☒	The statistical test(s) used AND whether they are one- or two-sided<br>*Only common tests should be described solely by name; describe more complex techniques in the Methods section.*
☐	☒	A description of all covariates tested
☐	☒	A description of any assumptions or corrections, such as tests of normality and adjustment for multiple comparisons
☐	☒	A full description of the statistical parameters including central tendency (e.g. means) or other basic estimates (e.g. regression coefficient) AND variation (e.g. standard deviation) or associated estimates of uncertainty (e.g. confidence intervals)
☐	☒	For null hypothesis testing, the test statistic (e.g. *F*, *t*, *r*) with confidence intervals, effect sizes, degrees of freedom and *P* value noted<br>*Give P values as exact values whenever suitable.*
☒	☐	For Bayesian analysis, information on the choice of priors and Markov chain Monte Carlo settings
☒	☐	For hierarchical and complex designs, identification of the appropriate level for tests and full reporting of outcomes
☐	☒	Estimates of effect sizes (e.g. Cohen's *d*, Pearson's *r*), indicating how they were calculated

Our web collection on statistics for biologists contains articles on many of the points above.

Software and code

Policy information about availability of computer code

Data collection	Google Trends (https://trends.google.com/trends) dataset was collected with pytrends (https://pypi.org/project/pytrends/, version 4.7.3) interface for automating downloading of reports from Google Trends, and calibrated using Google Trends Anchor Bank library (https://github.com/epfl-dlab/GoogleTrendsAnchorBank, version 0.5).
Data analysis	Data analysis was performed using python, version 3.8. Open-source libraries were used for data analysis (matplotlib v3.1.3, pandas v1.0.3, numpy v1.18.1, scikit-learn v0.22.1 scipy v1.4.1 statsmodels v0.11.0).<br><br>There are no restrictions on code availability. All the analysis code is publicly available in a GitHub repository: https://github.com/kristinagligoric/dietary-interests-covid.

For manuscripts utilizing custom algorithms or software that are central to the research but not yet described in published literature, software must be made available to editors and reviewers. We strongly encourage code deposition in a community repository (e.g. GitHub). See the Nature Portfolio guidelines for submitting code & software for further information.

Data

Policy information about availability of data

All manuscripts must include a data availability statement. This statement should provide the following information, where applicable:

- Accession codes, unique identifiers, or web links for publicly available datasets
- A description of any restrictions on data availability
- For clinical datasets or third party data, please ensure that the statement adheres to our policy

The data generated in this study is publicly available at https://github.com/kristinagligoric/dietary-interests-covid. The release at the time of submission was

archived in https://doi.org/10.5281/zenodo.5805181.

Field-specific reporting

Please select the one below that is the best fit for your research. If you are not sure, read the appropriate sections before making your selection.

☐ Life sciences ☒ Behavioural & social sciences ☐ Ecological, evolutionary & environmental sciences

For a reference copy of the document with all sections, see nature.com/documents/nr-reporting-summary-flat.pdf

Behavioural & social sciences study design

All studies must disclose on these points even when the disclosure is negative.

Study description	This is a quantitative observational study.
Research sample	Google search engine users in 18 countries. Study sample is chosen to achieve geographic diversity, and comprise countries with a large number of Internet users across continents: Brazil, Canada, Mexico, United States, France, Germany, Italy, Spain, United Kingdom, India, Indonesia, Japan, Egypt, Kenya, Nigeria, and Australia. Additionally, to achieve a varying severity of lockdowns, Sweden and Denmark were added as contrasting cases. Search engine users are not a representative sample of global population. Google Trends search interest (https://trends.google.com/trends) dataset was collected with pytrends (https://pypi.org/project/pytrends/, version 4.7.3) interface for automating downloading of reports from Google Trends, and calibrated using Google Trends Anchor Bank library (https://github.com/epfl-dlab/GoogleTrendsAnchorBank, version 0.5).
Sampling strategy	No sampling is performed as all Google search engine users contribute to aggregate search volume statistics published by Google.
Data collection	Google Trends (https://trends.google.com/trends) dataset was collected with pytrends (https://pypi.org/project/pytrends/, version 4.7.3) interface for automating downloading of reports from Google Trends, and calibrated using Google Trends Anchor Bank library (https://github.com/epfl-dlab/GoogleTrendsAnchorBank, version 0.5). The dataset was collected by sequentially obtaining search interest time series, for each of the studied foods, in each of the locations. Data collection was independent of the study hypotheses and conditions. No third-party was present during the data collection.
Timing	The dataset was collected between 9 February 2021 and 26 March 2021 (Australia, Brazil, UK, USA, Italy, India, France, Spain, Denmark, Mexico, Canada, Germany), and between 25 October 2021 and 2 November 2021 (Nigeria, Kenya, Indonesia, Egypt, Sweden, Japan).
Data exclusions	No data were excluded.
Non-participation	We rely on aggregated statistics. No users are dropped out.
Randomization	There was no random allocation. Covariates are controlled through regression discontinuity design modeling.

Reporting for specific materials, systems and methods

We require information from authors about some types of materials, experimental systems and methods used in many studies. Here, indicate whether each material, system or method listed is relevant to your study. If you are not sure if a list item applies to your research, read the appropriate section before selecting a response.

Materials & experimental systems

n/a	Involved in the study	
☒	☐	Antibodies
☒	☐	Eukaryotic cell lines
☒	☐	Palaeontology and archaeology
☒	☐	Animals and other organisms
☒	☐	Human research participants
☒	☐	Clinical data
☒	☐	Dual use research of concern

Methods

n/a	Involved in the study	
☒	☐	ChIP-seq
☒	☐	Flow cytometry
☒	☐	MRI-based neuroimaging
