# Supplementary material for: Population-scale dietary interests during the COVID-19 pandemic
Source: Nat Commun. 2022 Feb 28;13:1073. doi: 10.1038/s41467-022-28498-z (PMC8885865; doi:10.1038/s41467-022-28498-z)
Supplement: Supplementary file 4 — Description of Additional Supplementary Files [file 41467_2022_28498_MOESM4_ESM.pdf]

Title: Supplementary Data 1

Description: Entity-level Spearman rank correlation between interest and mobility. For modes, all entities are shown. For foods, top 10 entities most and least correlated across countries on average are shown (* marks $p<0.05$ between seasonality adjusted interest and mobility according to two-sided test with no correlation null hypothesis, - marks too low search interest in a country. All entities related to consuming food at home are correlated positively on average, all entities related to consuming food outside of home are correlated negatively on average, except barbecue.
